# New scenarios of *Trypanosoma cruzi* transmission in the Orinoco region of Colombia

**DOI:** 10.1590/0074-02760140403

**Published:** 2015-05

**Authors:** Lina María Rendón, Felipe Guhl, Juan Manuel Cordovez, Diana Erazo

**Affiliations:** 1 Departamento de Ciencias Biológicas, Centro de Investigaciones en Microbiología y Parasitología Tropical, Universidad de los Andes, Bogotá, Colombia; 2 Grupo de Investigación en Biología Matemática y Computacional, Departamento de Ingeniería Biomédica, Universidad de los Andes, Bogotá, Colombia

**Keywords:** Rhodnius prolixus, Chagas disease, Attalea butyracea, infestation index, infection index, insect domiciliation

## Abstract

Rhodnius prolixus, a blood-sucking triatomine with domiciliary anthropophilic habits, is the main vector of Chagas disease. The current paradigm of Trypanosoma cruzi transmission in Columbia includes a sylvatic and domiciliary cycle co-existing with domestic and sylvatic populations of reservoirs. The aim of this study is to evaluate the population densities and relative abundance of triatomines and mammals that may be involved in the sylvatic cycle of Chagas disease to clarify the epidemiological scenario in an endemic area in the province of Casanare. Insect vectors on Attalea butyracea palms were captured using both manual searches and bait traps. The capture of mammals was performed using Sherman and Tomahawk traps. We report an infestation index of 88.5% in 148 palms and an index of T. cruzi natural infection of 60.2% in 269 dissected insects and 11.9% in 160 captured mammals. High population densities of triatomines were observed in the sylvatic environment and there was a high relative abundance of reservoirs in the area, suggesting a stable enzootic cycle. We found no evidence of insect domiciliation. Taken together, these observations suggest that eco-epidemiological factors shape the transmission dynamics of T. cruzi, creating diverse scenarios of disease transmission.

The transmission main mode of the parasite *Trypanosoma cruzi*, which causes Chagas disease, is through a vector (WHO 2002). Twenty-six species of triatomines have been reported in Colombia, of which 15 have been found to be naturally infected with* T. cruzi.* In countries such as Colombia and Venezuela, the main vector of the disease is *Rhodnius prolixus*, a species of triatomine that has a wide geographical distribution and presents domiciliary anthropophilic habits (Pinto et al. 2006 ). Studies in the Orinoco region of Colombia and Venezuela have shown that populations of *R. prolixus* are a risk factor for the transmission of *T. cruzi* to humans (Sanchez-Martin et al. 2006, Fitzpatrick et al. 2008, Guhl et al. 2009 ). Indeed, vector transmission in Colombia represents a public health problem in both the Orinoco region (Arauca, Casanare) and the provinces of Norte de Santander and Santander (Guhl et al. 2007). Studies in Casanare have revealed parasite transmission characterised by high rates of *R. prolixus* infestation of palms next to dwellings and *R. prolixus* has been shown to be well adapted to domicile habitats in this region (Angulo et al. 2012). In addition, a recent study conducted in Casanare reports sylvatic *R. prolixus* populations associated with both native palms (*Attalea butyracea*) and introduced, agro-industrial palms (*Elaeis guineensis*). The natural infection indexes for *T. cruzi* found in insects caught in these palm species were 67% and 41%, with infestation indexes of 92.8% and 100%, respectively (Guhl et al. 2007). However, there are few reports about the co-occurrence of domestic and sylvatic cycles and their stabilities and the ecological characteristics of the mammalian reservoirs involved in the transmission of *T. cruzi*.

Vector dispersion between domestic and wild habitats may occur both actively, when insects are attracted to houses by light and passively, for example, when palm leaves are used to build the roofs of rural dwellings in endemic areas (Guhl et al. 2009). Triatomine reinfestation of houses by wild populations of *R. prolixus* indicates the ineffectiveness of conventional control strategies (Fitzpatrick et al. 2008) and previous studies in 109 villages in Casanare found a reinfestation index of 18% in dwellings after two cycles of spraying (Guhl et al. 2009). Nonetheless, effective vector control methods, such as implementing home improvements and the use of bed nets and window nets impregnated with residual insecticides, can reduce the risk of reinfestation and eliminate contact between insects and the dwelling inhabitants. Accordingly, the use of cost-effective monitoring strategies depends on an understanding of such aspects as the population densities and reproductive cycles of vectors and reservoirs, the infestation indexes of palms growing close to dwellings and the infection indexes of triatomines (Dias et al. 2002).

This paper reports the mammalian species abundance, insect population densities and absence of a domestic cycle of an area with active parasite transmission. The results challenge the current paradigm of disease transmission characterised by co-existing sylvatic and domiciliary cycles. These findings could promote useful control measures for different epidemiological transmission scenarios.

## MATERIALS AND METHODS

*Study area* - Casanare is an area of great interest because it has all of the features associated with the dynamics of the transmission cycle of Chagas disease. In this region, there are reports of 100% infestation indexes in palms, a natural infection index of 67% in triatomines and a *T. cruzi* infection index of 89% in *Didelphis marsupialis *(Guhl et al. 2009).

The village of El Amparo in the town of Maní was selected because of reports of high *R. prolixus *infection indexes of *T. cruzi* (Pinto et al. 2005, 2007, Guhl 2010). Additionally, this municipality is situated close to plantations of African oil palm (*E. guineensis*), where a high triatomine infestation index has been reported (Guhl et al. 2005). Maní has a total area of 3,860 km^2^ and 11,150 inhabitants; the town is located at 200 m above sea level and has a warm and humid climate with an average temperature of 27ºC. This municipality has two seasons: summer, during the months of November-March and winter, from late April-October.

*Triatomine collection* - An active search for triatomines in the selected area was conducted in palms near houses; in total, 148 properly georeferenced *A. butyracea* palms were selected in an area of approximately 100 ha (Figure). To determine the density of *R. prolixus* in *A. butyracea, *insects were captured using both manual searches and bait traps. The manual searching involved a 1 h thorough exploration of the palm's crowns by an experienced field biologist. In addition, a white sheet was placed at the base of the tree for inspection of any material released from leaves that were detached. For the bait trapping (Angulo & Esteban 2011), each trap was strategically placed at 05:00 pm at the top of palms that had previously been manually sampled. The traps were left overnight and collected early in the morning on the next day.

**Figure:**
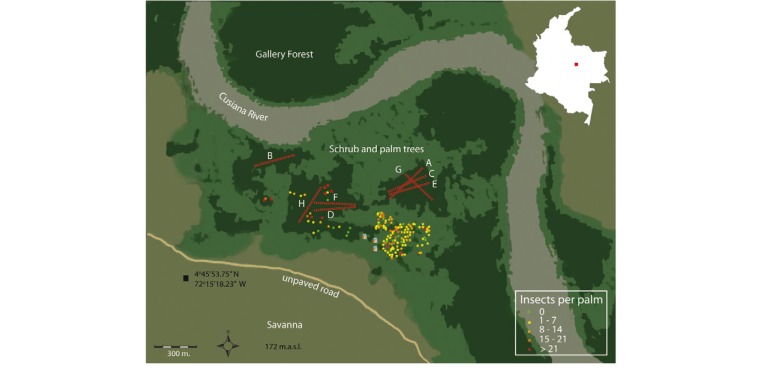
The study area is located near the small town of Maní, Casanare, Colombia (see red dot in upper right corner to locate the area in Colombia) in the low valley of the Cusiana River [172 m above sea level (m.a.s.l)]. The region is characterised by warm weather (27ºC), one rainy season (average of 2,714 mm of rainfall per year) and it is covered mainly by savanna and gallery forest. The main economic activity is cattle, oil palms and oil drilling. Eight transects (A-H, represented by red dashed lines) about 300 m long were set in area of about 130 ha comprising three distinct types of ecotopes: gallery forest (dark green), shrubs and palms (light green) and savanna (olive green). Stars represent the location of palm trees where insects were collected and are colour-coded based on densities (see inset on the right bottom corner). Small icons represent houses. Only three houses were included in the study area but 36 additional dwellings were sampled for insects in the close village of Chavinave.

The search for triatomines inside dwellings was conducted in 39 houses (3 in the study area and 36 in the small village of Chavinave, 10 km away) using Gómez-Nuñez's (1965) boxes located in various sites of the houses and checked every three months for a period of eight months. The captured insects were collected and labelled with geographical coordinates obtained by GPS, the palm's code and the date of capture. The sampling data from all the methods, i.e., in the palm trees using manual searches and bait traps and within the houses, were recorded in a format that included all of the information from both the capture site and the collected specimens (number of insects per palm, sex, species, stage).

*T. cruzi infection in triatomines* - Only collected insects from the first to the fourth instar were brought to the laboratory; these specimens were maintained until adulthood by feeding on mice in an insectary to obtain leg samples of each for population genetic analysis using microsatellite markers (unpublished observations). Adults and fifth-instar individuals were examined on-site for the detection of *T. cruzi* infection and carefully dissected to obtain samples of legs, body, faeces and intestinal contents. The insects were washed in White's solution (HgCl_2_, NaCl, HCl, ethanol, distilled water) for 10 min to reduce bacterial contamination and then placed in a 0.9% saline solution for 2 min. Faeces were then removed by abdominal massage. The faecal sample was dissolved in a saline solution and observed by direct microscopy. A 0.5 mL aliquot was added to a blood culture for the detection of trypanosomes. Additionally, the rectal ampulla with the intestinal content was removed and stored volume:volume in a guanidine solution (Guanidine chloride, EDTA, pH 8.0) for subsequent analysis. The data for each individual (capture code, stage, sex, infection) was recorded.

*Capture of mammals* - Mammals were captured in two different ecotopes in the study area. Four linear transects of approximately 300 m were established in each ecotope, with 30 capture points, to determine the relative species abundance (Figure). Twenty aluminium Sherman traps (10 cm x 11 cm x 38 cm) with a locking pressure-driven door in the inner floor and 10 Tomahawk traps (17 cm x 17 cm x 50 cm) with the same seal were placed in each transect. Two Sherman traps were placed for each Tomahawk trap. Additionally, three large Tomahawk traps (45 cm x 45 cm x 110 cm) were placed at the beginning, middle and end of each transect. Each baited trap was placed at sites with no direct sunlight and was protected from rain. All transects were labelled with an alphabetical system (A-H) and numbered sequentially at each capture point (1-30). Each capture point was georeferenced using GPS and marked with tape for the ensuing captures at different periods of the year. This type of identification of each transect allows an accurate record of the location of the captures and suitable release of a trapped animal at the same location. Collection in each transect was performed for five nights. The transects were checked daily in the morning and any captured animals were identified with the number of point of capture. Animal traps that were removed from the transect were replaced with new ones and the one containing the animal was placed in a dark cloth bag before being transported to the field laboratory to avoid any contact or risk of infection and to reduce animal stress. The animals transported to the laboratory were sampled as soon as possible.

The capture of bats was carried out manually in the tops of the palm trees at the same time as the capture of triatomines. The captured animals were placed in small cloth bags until further processing.

Each captured individual was immobilised with an intramuscular injection of anaesthetic (Zoletil^(r)^ 50 - Virbac Laboratories, Mexico), with an approximate dose of 10 mg/kg on the outside of one hind limb. Small animals captured in Sherman traps, e.g., rodents, were moved to cloth bags to be anesthetised. Medium-sized mammals, such as marsupials, were anesthetised directly in the trap and, once asleep, were carefully removed to begin the sampling process. Blood samples were collected aseptically by cardiac puncture from the wild reservoirs captured and each specimen was marked on the ear to allow reidentification if recaptured.

The georeferenced sites of capture were described in record format with all the information of the specimen (size, sex, estimated body mass, reproductive status, hair colour, teeth, biometrics). Photographic records were also obtained for each specimen for subsequent identification. After sampling and full recovery of the animal, each individual was released at the same point where it was captured.

Blood samples were also taken from dogs (*Canis lupus familiaris*) from the dwellings where insect traps were placed. The anaesthesia and sampling procedures used were the same as for the animals caught in traps; the blood sample was taken from one of the front legs.

The methods of animal capture and handling followed the recommendations of the capture and handling guide approved by the Committee of the American Society of Animal Care and Use of Mammals (Gannon & Sikes 2007). Additionally, according to the Act 134 of 2011 of the Ethical Committee for Research of the University of Los Andes, the project accomplished all scientific, technique and administrative rules for health research established in Resolution 008430 of 1993 of the Ministry of Health.

*T. cruzi infection in mammals* - The blood sample from each individual was initially examined by direct microscopy and distributed into two-phase blood culture tubes (solid medium with liver infusion tryptose) for the maintenance of the parasite in the event that the reservoir was infected. Due to the low sensitivity of the haemoculture technique, the samples were also stored in two 1.5 mL Eppendorf tubes with guanidine (volume:volume) for subsequent molecular analyses of genotyping and food sources (unpublished observations). Approximately 0.5 mL of the sample from each tube was used. Blood cultures were kept at room temperature until transport to the laboratory, where they were reviewed every 20 days for six months. Positive blood cultures were maintained until a number of abundant forms were achieved by direct microscopy and were then cryopreserved.

## RESULTS

*Infestation index in palms* - A total of 1,417 insects were captured in the study. Using Lent and Wygodzinsky (1979) morphological key, all insects were initially identified as *R. prolixus*. Of the 1,417 insects captured, only 269 were adults or fifth-stage nymphs. However, due technical and economical limitations, a random sample of 85 of the 269 total were analysed using genetic markers (CitB and AmpG) and confirmed to be *R. prolixus*. Samples collected from each individual were used for population analysis using microsatellite markers, the determination of food sources by high resolution melting and the molecular characterisation of *T. cruzi* discrete typing units (data not shown). A total infestation index of 88.5% in *A. butyracea* palms was found.

In the sampled dwellings, nine adult insects were manually collected near light bulbs; these insects most likely originated from palms close to the domicile. All the traps were empty and free of insect vestiges every time they were checked.

*Infection indexes in triatomines* - Of the 269 triatomines dissected, 70.3% were found to be adults; the remaining 29.7% were fifth-instar individuals. A natural infection index of 60.2% was found. The first to fourth-stage nymphs captured were maintained in an insectary for feeding and growth and subsequent parasite detection. The *T. cruzi* natural infection index in the insects captured in the dwellings was 55.5%.

*Infection index in mammals* - The 160 mammals reported in Table were captured in eight transects. The average number of mammals captured per transect was 14, with no difference found in the number of individuals captured per transect. Of the 160 mammals that were caught and later released, 19 blood cultures were positive for *T. cruzi* infection, corresponding to an infection index of 11.9% (Table). Positive individuals belonged to the following species: *D. marsupialis*, *Tamandua tetradactyla*, *Proechimys oconnelli*, *Marmosa andersoni* and *Artibeus lituratus*.

**TABLE:** Data of mammals captured in eight transects (A-H)

Mammals captured (order)	A	B	C	D	E	F	G	H	P	Individuals (n)	Infection index (%)
Didelphimorphia											
*Marmosa andersoni*	9 (2)	5	8 (1)	4	6 (2)	13	1	6	-	52	10
*Didelphis marsupialis*	4 (1)	0	4 (2)	6 (1)	2 (1)	2	2	4	-	24	21
Pilosa											
*Tamandua tetradactyla*	0	0	0	1 (1)	1 (1)	0	0	0	-	2	100
Rodentia											
*Proechimys oconnelli*	2	2	2	0	1	1	0	1	-	9	0
*Heteromys anomalus*	0	1	0	0	0	0	0	1	-	2	0
*Dasyprocta fuliginosa*	0	1	1	0	0	0	0	0	-	2	0
Unidentified	5 (1)	4 (1)	6 (1)	2	0	1	4	1 (1)	-	23	17
Chiroptera											
*Artibeus lituratus*	-	-	-	-	-	-	-	-	7 (1)	7	14
Uroderma	-	-	-	-	-	-	-	-	7	7	0
Unidentified	-	-	-	-	-	-	-	-	32 (2)	32	6
Individuals	20	13	21	13	10	17	7	13	46		
Infection index per transect (%)	20	8	19	15	4	0	0	8		160	12

bats were collected from palms only (column label P). Several species were identified, others were grouped at the order level. Numbers in brackets are the number of individuals naturally infected with Trypanosoma cruzi per specie per transect

Seventeen dogs (*C. l. familiaris*) were sampled as domestic reservoirs. None of the samples tested positive for *T. cruzi* infection, either by direct microscopy or by blood culture.

## DISCUSSION

The area in which this study was performed is important for Chagas disease transmission in Colombia. First, it is known that the region has high prevalence of triatomines in *A. butyracea* palms (Pinto et al. 2005, Guhl et al. 2007, 2009). Second, *A. butyracea *is widely distributed in the country and a similar species (*E. guineensis*) is being grown in the area for economic purposes; thus, the results of this study could promote similar investigations in other regions. Third, the use of palm leaves for roofing in rural dwellings and the use of the fruit for making juice and wine increase the risk of vectorial and oral infection. Finally, in the Eastern Plains (Casanare, Arauca, Vichada, Meta, Vichada and Guaviare areas), which covers more than 150,000 km^2^ (Botiva et al. 1989), *A. butyracea* forms large populations in gallery forests along streams (Galeano & Bernal 2010) and provides shelter for a wide range of mammals, birds and reptiles. Many of the mammals found in the tops of the palms, such as marsupials, vermilinguans and bats, serve as a food source for triatomines and as potential reservoirs of the parasite; these mammal populations play an important role in the transmission dynamics of the sylvatic cycle of Chagas disease. Recently, an acute oral outbreak of Chagas disease occurred in the town of Paz Ariporo (Casanare), affecting 31 workers from companies linked to the mining sector who were exposed to food contaminated with either traces or the faeces of infected triatomines.

Of the 1,417 insects captured, 269 were dissected and a subgroup of 85 was characterised as *R. prolixus*. We plan to continue the identification of all the collected insects, but this preliminary result is interesting because *Rhodnius robustus* (a triatomine species with sylvatic habits and morphologically very similar to *R. prolixus*) is also expected to be distributed in the area (Lent & Valderrama 1973, Monteiro et al. 2003). This observation is important for two reasons. (i) *R. robustus* has a poor domiciliation capacity compared to *R. prolixus*, which makes *R. prolixus* a more competent vector (Monteiro et al. 2003). Thus, from an epidemiological perspective, palm infestation with *R. prolixus* proposes a riskier scenario. (ii) In the present study, we did not find evidence of domiciliation in *R. prolixus*, suggesting that the sylvatic and domestic preferences of these two related species need to be revised.

In Colombia, *R. prolixus* has been found in three species of palms in the Eastern Plains, with the highest percentage of infestation found in *A. butyracea *(D'Alessandro et al. 1981). Several studies have reported infestation indexes of 64.53-100% in *A. butyracea* in Casanare (Guhl et al. 2007, 2009, Angulo et al. 2012). The infestation index found in the present study (88.5%) confirms that *A. butyracea* is one of the main shelters for *R. prolixus* and is consistent with the infestation index of 83.33% reported in a previous study (Angulo et al. 2013) The number of triatomines per palm found in the area (1,417 insects in 148 palms - 9.57 insects per palm) is significantly less compared to a previous report in which more than 2,000 insects were found in 102 palms (> 19.6) in Casanare (Pinto et al. 2005). High infestation indexes suggest that entomological surveillance could be important for reducing the risk of human infection.

An interesting finding is that fewer triatomines were collected in the rainy season (data not shown) compared to summer, during which much higher triatomine population densities were found in the palm trees. Little information is known about the impact of climatic conditions and the population biology of *R. prolixus*. Although a study by Luz et al. (1999) showed that constant and fluctuating factors such as humidity and temperature affect the development of this insect, there is a need to investigate this issue in more detail.

The natural infection index of 60.2% found in triatomines corroborates the infection indexes reported (≈67%) in different areas of the department that have active transmission of the parasite (Guhl et al. 2007, 2009). Considering that a large percentage of the insects found in palms near dwellings are infected, knowledge of the infection levels of triatomines is crucial for proposing control strategies to avoid contact between vectors and humans.

Most of the mammals (81.2%) were captured in November, March and early April. The remaining 18.8% was captured in the rainy season, in May; as with the insects, this result could be explained in terms of food availability and the breeding cycle. The infection index in mammals (11.9%) was low compared to the infection index observed in triatomines, but their relative importance to disease transmission risk to humans needs to be weighted in terms of their abundance, life expectancy, body size and immune response to the parasite.

Over 180 species of domestic, synanthropic and sylvatic mammals, primarily rodents and opossums have been reported to be infected with *T. cruzi *(Noireau et al. 2009). In the present study, 13% of the marsupials (*M. andersoni* and *D. marsupialis*) were infected with *T. cruzi* compared to 10% infection for placentals. In addition, rodents showed a natural infection of 11.11%. To our knowledge, studies investigating infection indexes in mammals have been performed only in *D. marsupialis*. Previous reports found infection indexes between 57-89% (Travi et al. 1994, Pinho et al. 2000, Pinto et al. 2005, de Lima et al. 2006), considerably higher compared to that found in our study. These differences could be explained in part by the different methods used to determine infection. We used haemoculture in combination with direct microscopy, a combination that increases sensitivity. Due to the low sensitivity of the haemoculture technique, the samples were also stored in guanidine for subsequent molecular analyses of genotyping and food sources (unpublished observations). Given that *D. marsupialis* is abundant, widely distributed and a synanthropic species, we consider that further examination of this topic is of particular relevance. Indeed, given that the parasite can multiply extracellularly in the scent glands, *D. marsupialis* is proposed to be not only a reservoir, but also a vector of *T. cruzi* (Jansen et al. 1999).

No eggs or nymphal stages, which are an indication of the domiciliation of triatomines, were found inside the human dwellings. This observation may indicate that the sylvatic cycle of the parasite is robust and that the insects found in the houses were most likely attracted by light and flew into the domicile from nearby palm trees. Our use of "robust" indicates that the sylvatic populations of *R. prolixus* can maintain an enzootic cycle with terrestrial and arboreal mammals, which supply all the food requirements for the insect's development (Noireau et al. 2009). The migration of insects from nearby palms to houses could be due to intrusion, a phenomenon described for several species of triatomines in several countries, including Colombia (Angulo et al. 1999, Texeira et al. 2001).

We found that 80% of the insects collected in palms were nymphs and concluded that the biological and ecological conditions in palms appear to provide a proper shelter and appropriate reproductive conditions for insect vectors. It is important to stress the fact that *A. butyracea *is the main wild ecotope for these triatomine populations and an ecological marker for potentially identifying the distribution of vectors and reservoirs of the *T. cruzi *sylvatic cycle (Romaña et al. 1999).

It has been shown in other regions, such as the Andean piedmont and inter-Rift Valley, that *R. prolixus* has a strong tendency to infest dwellings (Guhl et al. 2007), yet the current study provides an example of *R. prolixus* populations that are stable in their wild ecosystem. To investigate whether a prominent sylvatic cycle co-exists with high human prevalence, we conducted a small study in 36 houses in a village called Chavinave (in addition to the 3 houses that are within the study area), 10 km away. The locale is characterised by a higher house density compared to the study area and fewer proximal palms. Gómez-Nuñez (1965) traps were placed in several locations of the houses and left for a period of eight months (as described in the Materials and Methods section). No insects were found in any of the traps, confirming the scenario of a lack of triatomine domiciliation in the area.

A small serological study of school children in the villages of Chavinave and el Amparo [conducted in parallel with our study by the Health Promotion Institute (IPS) Servinsalud, Casanare] found 2% *T. cruzi* infection. Sampling was carried out on 79 children aged zero-15 years, with only one child from El Amparo testing positive according to two different techniques: ELISA and indirect fluorescence immunoassay (informed consent was obtained according to the regulations of the IPS Servinsalud, Yopal, Casanare). These serological results are in agreement with the hypothesis of no-domiciliation and a robust sylvatic cycle.

We concluded from this study that diverse scenarios of *T. cruzi* transmission could be present in different geographical regions based on their ecological, climatological, epidemiological and sociological characteristics. In particular, the Casanare region was thought to be a high endemic area with the co-existence of domiciliary and sylvatic *T. cruzi* cycles. The results from this region suggest no evidence of domiciliation and a robust sylvatic cycle.
